# Poster Session II – Poster of Distinction II - A206 EXPANSION OF PERIPHERAL RORγT^+^ POLYMORPHONUCLEAR MYELOID-DERIVED SUPPRESSOR CELLS (PMN-MDSCS) SIGNALS EARLY IMMUNE DYSREGULATION LINKED TO CROHN’S DISEASE RISK

**DOI:** 10.1093/jcag/gwaf042.206

**Published:** 2026-02-13

**Authors:** B Thakur, B Bharali, D Turner, A Griffiths, R Panaccione, S Murthy, H Steinhart, K Jacobson, A Martin, S Lee, W Turpin, K Croitoru

**Affiliations:** University of Toronto, Toronto, ON, Canada; Lunenfeld Tanenbaum Research Institution, Sinai Health, Toronto, ON, Canada; Shaare Zedek Medical Center Children, Israel, Israel; SickKids Research Institute Peter Gilgan Centre for Research and Learning, Toronto, ON, Canada; University of Calgary, Calgary, AB, Canada; Ottawa Hospital Research Institute, Ottawa, ON, Canada; Lunenfeld Tanenbaum Research Institution, Sinai Health, Toronto, ON, Canada; BC Children’s Hospital, Vancouver, BC, Canada; University of Toronto, Toronto, ON, Canada; Lunenfeld Tanenbaum Research Institution, Sinai Health, Toronto, ON, Canada; Lunenfeld Tanenbaum Research Institution, Sinai Health, Toronto, ON, Canada; Lunenfeld Tanenbaum Research Institution, Sinai Health, Toronto, ON, Canada

## Abstract

**Background:**

Crohn’s disease (CD) is a chronic inflammatory bowel disease thought to be driven, in part, by an exaggerated immune response to intestinal microbes. While impaired epithelial barrier function, and anti-microbial antibodies can be detected years before CD onset; the immunological changes preceding CD onset remains unexplored

**Aims:**

To identify immune signatures associated with CD risk by phenotyping the peripheral immune landscape during the preclinical phase of CD

**Methods:**

In a nested case-control cohort of the CCC-GEM Project, which prospectively follows healthy first-degree relatives (FDRs) of CD patients, 80 FDRs who developed CD (pre-CD) were matched by age, sex, follow-up duration, and location with FDRs who remained healthy (HMC, n = 310). PBMCs collected at recruitment were analyzed by single-cell mass cytometry using 40 phenotypic and functional markers. Immune subsets were identified by automated unbiased clustering, and their associations with future CD, faecal calprotectin (FCP), serum proteomics (Olink®), and antimicrobial antibodies (Prometheus®) were assessed using conditional logistic regression, generalized estimating equations, or partial Spearman correlation as appropriate. ROC analyses evaluated performance of immune subsets to stratify future CD cases from controls

**Results:**

A distinct peripheral immune landscape, characterized by increased myeloid and reduced lymphoid subsets, was associated with FCP and future CD (p = 0.00003-0.05). Subgroup analysis based on median follow-up time to diagnosis revealed temporal immune alterations during the preclinical phase, with early expansion of RORγt^+^ PMN-MDSCs and CCR9^+^ dendritic cells occurring more than 2.5 years prior to CD onset, preceding the peripheral loss of α4β7^+^ activated naïve-like T and B cells observed within 2.5 years before diagnosis. Early expansion of these myeloid subsets correlated with elevated serum ASCA- or ompc-IgA levels. Although RORγt^+^ PMN-MDSCs remained elevated until CD onset, proteomic analysis revealed a shift from regulatory to proinflammatory phenotypes approaching CD onset. Finally, ROC analysis identified RORγt^+^ PMN-MDSCs as having the highest potential for CD risk stratification (AUC=0.821).

**Conclusions:**

This study delineates the immune cell alterations associated with future CD and identifies RORγt^+^ PMN-MDSCs as a potential early immune marker for CD risk stratification and target for preventive intervention.

**Funding Agencies:**

CCC

